# Luteoloside Inhibits IL-1β-Induced Apoptosis and Catabolism in Nucleus Pulposus Cells and Ameliorates Intervertebral Disk Degeneration

**DOI:** 10.3389/fphar.2019.00868

**Published:** 2019-08-05

**Authors:** Jialiang Lin, Jiaoxiang Chen, Zengjie Zhang, Tianzhen Xu, Zhenxuan Shao, Xiaobin Wang, Yuanzhe Ding, Naifeng Tian, Haiming Jin, Sunren Sheng, Weiyang Gao, Yan Lin, Xiaolei Zhang, Xiangyang Wang

**Affiliations:** ^1^Department of Orthopaedics, The Second Affiliated Hospital and Yuying Children’s Hospital of Wenzhou Medical University, Wenzhou, China; ^2^Zhejiang Provincial Key Laboratory of Orthopaedics, Wenzhou, China; ^3^The Second School of Medicine, Wenzhou Medical University, Wenzhou, China; ^4^Department of Orthopaedics, The Third Affiliated Hospital and Ruian People’s Hospital of Wenzhou Medical University, Ruian, China; ^5^Chinese Orthopaedic Regenerative Medicine Society, Hangzhou, China

**Keywords:** intervertebral disk degeneration, luteoloside, apoptosis, Nrf2, inflammation, nucleus pulposus cell

## Abstract

Intervertebral disk degeneration (IDD) is the major cause of low back pain (LBP), which affects 80% of the world’s population. Interleukin 1 beta (IL-1β) is a major inflammatory factor that accelerates disk degeneration, and IL-1β levels increase in degenerative disks. It has recently been reported that luteoloside—a type of flavonoid glycoside—has anti-inflammatory properties. In the present study, we investigated the protective potential of luteoloside in IDD. We found that luteoloside maintains cell morphology and inhibits apoptosis (indicated by the reduced expression of cleaved caspase 3) in IL-1β-treated nucleus pulposus (NP) cells. It also suppresses inflammatory mediators—nitric oxide (NO), prostaglandin E2 (PGE2), tumor necrosis factor alpha (TNF-α), interleukin 6 (IL-6), cyclooxygenase 2 (COX-2), and inducible nitric oxide synthase (iNOS)—in IL-1β-treated NP cells. Furthermore, we found increased collagen II and aggrecan expression and reduced MMP13 and ADAMTS5 expression in luteoloside-treated NP cells in the presence of IL-1β. Nuclear factor (erythroid-derived 2)-like 2 (Nrf2) is involved in apoptosis, inflammation, and extracellular matrix (ECM) homeostasis. Mechanistic studies revealed that the NF-κB signaling pathway is inhibited by luteoloside, and Nrf2 is involved in the regulation of luteoloside in NF-κB signaling because Nrf2 knockdown reduced the suppressive effect of luteoloside on NF-κB signaling. We also established a puncture-induced rat IDD model and demonstrated that the persistent intraperitoneal injection of luteoloside ameliorates the progression of IDD. In conclusion, we demonstrated that luteoloside activates the Nrf2/HO-1 signaling axis and is a potential therapeutic medicine for IDD.

## Introduction

Low back pain (LBP) is a common disorder of the musculoskeletal system throughout the world and is responsible for years of disability in most countries and age groups (Luoma et al., 2000; Jensen et al., 2017). It was ranked the fourth leading cause of disability-adjusted life years (DALYs) globally in 2015 (Hurwitz et al., 2018). Over half a billion people worldwide have LBP, and approximately 80% of individuals experience it at some point in their life (Vos et al, 2017). Prevention and therapeutic intervention are hampered because the exact cause of LBP remains unclear; however, a correlation between LBP and intervertebral disk degeneration (IDD) has been documented (Scheele et al., 2012; Wang et al., 2012; Yelin et al., 2016). IDD is considered one of the key predisposing factors for LBP (Lutz et al., 2003).

Intervertebral disks are avascular organs that comprise three tissues: the nucleus pulposus (NP), the annulus fibrosus (AF), and the endplates (EP), which are located on the upper and lower sides of the NP (Vergroesen et al., 2015). The gelatinous NP is the main functional component of the intervertebral disk and plays a vital role in distributing the load and maintaining the stability and flexibility of the spine (Chen et al., 2016). The loss of NP cells and the breakdown of extracellular matrix (ECM) components are believed to be important in IDD (Chen et al., 2017; Tu et al., 2017). During the development of IDD, multiple abnormal stimuli can increase the level of inflammatory cytokines—especially interleukin 1 beta (IL-1β) and tumor necrosis factor alpha (TNF-α)—which then promote the degradation of aggrecan and collagen, the generation of catabolic factors [e.g., matrix metalloproteinases (MMPs)], the leakage of NP cells, and changes to the phenotypes of the intervertebral disk cells (Risbud and Shapiro, 2014; Xu et al., 2017; Cheng et al., 2018). Several inflammatory mediators and catabolic factors are reportedly upregulated following IL-1β treatment. These include cyclooxygenase 2 (COX-2), prostaglandin E2 (PGE2), nitric oxide (NO), NO synthase (NOS), ADAMTS5, and MMPs, which impair the ECM (Tang et al., 2017). Because NP cells can produce cartilage-specific ECM, the apoptosis of NP cells induced by increased proinflammation cytokines may lead to the remodeling of various cell types and a reduction in ECM synthesis, thereby accelerating the pathological process of IDD (Choi et al., 2018; Wu et al., 2018). Therefore, reducing the inflammation response, inhibiting the apoptosis of NP cells (Zheng et al., 2018), and reversing the imbalance between anabolism and catabolism within the NP microenvironment are effective strategies for the treatment and prevention of IDD.

Luteoloside (Lut; also known as luteolin-7-*O*-beta-d-glucoside) can be extracted from the plant *Lonicera japonica*; it is a type of flavonoid glycoside and has recently been reported to possess anti-inflammation and antibacterial properties (Song et al., 2018). Luteoloside suppresses the inflammation response by inhibiting the degradation of nuclear factor-kB inhibitor (IkB), an indicator of the activation of nuclear factor-kB (NF-kB) (Hsuan et al., 2015), which is closely associated with IDD (Yao et al., 2016; Li et al., 2017). Nuclear factor (erythroid-derived 2)-like 2 (Nrf2) is involved in antioxidant defense and the cell survival response through a battery of cytoprotective genes in various degenerative diseases (Khan et al., 2017; Vaamonde-Garcia et al., 2017). It plays different roles in inflammation and apoptosis in various neurological disorders (Wang et al., 2017). Furthermore, previous studies have shown that inhibiting NF-κB *via* activation of the Nrf2/HO-1 signaling pathway protects NP cells and chondrocytes (Zhang et al., 2017; Khan et al., 2018; Zheng et al., 2018). Moreover, in previous studies, we found that luteoloside promotes the expression of Nrf2 in the nuclei of NP cells. However, its effect on NP cell dysfunction remains unclear. Thus, in the present study, we investigated the anti-inflammatory and anti-apoptosis effects of luteoloside on IL-1β-stimulated NP cells and explored the underlying mechanism.

## Material and Method

### Ethics Statement

This study was carried out in accordance with the principles of the Basel Declaration and the study was approved by Wenzhou Medical University (wydw2014-0129).

### Reagents and Antibodies

Luteoloside (C21H20O11) (purity>98%) was purchased from Chengdu Herbpurify Co., LTD (Chengdu, China). Dimethylsulfoxide (DMSO) and collagenase II were purchased from Sigma-Aldrich (St. Louis, MO, USA). The primary antibodies of β-actin, MMP13, collagen II, ADAMTS 5, aggrecan, Nrf2, HO-1, and lamin B1 were purchased from Abcam (Cambridge, UK). Second antibody, such as Alexa Fluor^®^488 labeled and Alexa Fluor^®^594 labeled Goat Anti-Rabbit IgG (H+L), was obtained from Abcam (Cambridge, UK). The cleaved-caspase 3, Bax and Bcl-2, iNOS, COX-2, IκBα, and p65 antibodies were obtained from CST (MA, USA). The 4’,6-diamidino-2-phenylindole (DAPI) was obtained from Beyotime (Shanghai, China).

### Surgical Procedure

Thirsty-six Sprague–Dawley rats (200–250 g) were randomly classified into three groups, including control group (*n* = 12), IDD group (saline injected after surgery, *n* = 12), and luteoloside group (luteoloside injected after surgery, *n* = 12). As described in the previous study (Chen et al., 2016), IDD group and luteoloside group rats were anesthetized by 2% (w/v) pentobarbital (40 mg/kg), and needles (27G) were used to puncture the whole layer of AF through the tail skin. All the needles were kept in the disk for 1 min. After surgery, the luteoloside solution was immediately injected intraperitoneally to deliver a dose of 10 mg/kg/day in luteoloside group, and the saline was injected in IDD group and control group every day until the rats were sacrificed.

## Isolation and Culture

### Primary Rat Nucleus Pulposus Cells

10 or more randomly selected SD rats were euthanized by an overdose of sodium pentobarbital. As described in the previous study (Chen et al., 2016), NP tissues were collected by a dissecting microscope. Then, the tissues digested by 2 mg/ml 0.1% collagenase II for 4 h at 37°C. Next, the digested NP tissues were transferred to DMEM/F12 (Gibco, Invitrogen, Grand Island, NY) with 15% fetal bovine serum (FBS; Gibco, Invitrogen, Grand Island, NY) and antibiotics (1% penicillin/streptomycin) in the incubator at 5% CO2 at 37°C. And the medium was firstly changed after incubation for 24 h, harvesting the NP cells by 0.25% Trypsin-EDTA (Gibco, Invitrogen) when confluent. Next, NP cells were passed into 10-cm culture plates at the appropriate density, about 120,000 cells a plate. The complete medium was changed every other day, and the first two and three passage NP cells were used in our experiments.

### Cell Viability Analysis

The cytotoxicity of luteoloside on NP cells was evaluated by Cell Counting Kit-8 (CCK-8; Dojindo Co., Kumamoto, Japan) referring to the protocol. As described in the previous study (Chen et al., 2016), NP cells were treated with luteoloside for 24h and washed by phosphate-buffered saline (PBS) for one time, and then 100 μl of DMEM/F12 containing 10 μl of CCK-8 solution was added to each well of the plate for 2 h at 37°C. Then, the OD was measured at 450 nm using a micro-plate reader. All experiments were performed in triplicate.

### Immunofluorescence

For immunofluorescence, as described in the previous study (Chen et al., 2016), the cells were washed by PBS for three times before fixation using 4% paraformaldehyde, followed by permeation using the 0.5% Triton for 5 min. Cells were blocked by 10% bovine serum albumin for 1 h at 37°C; cells were washed by PBS and incubated with primary antibodies, Nrf2 (1:200), collagen II (1:200), MMP-3 (1:200), and P65, in a humid chamber overnight at 4°C. On the next day, NP cells were incubated with Alexa Fluor^®^488/594 labeled conjugated second antibodies (1:400) for 1 h at 37°C, and nuclear of NP cells were labeled with DAPI for 5 min. Finally, each slide was observed with a fluorescence microscope (Olympus Inc., Tokyo, Japan), and the fluorescence intensity was quantified by Image J software 2.1 (Bethesda, MDUSA) by double-blinded observers who were blinded to the experimental groups.

### Western Blot Assay

The total protein of NP cells was extracted using RIPA lysis buffer containing with 1 mM PMSF (phenylmethanesulfonylfluoride), and concentration of extracted protein was measured using the BCA Protein Assay Kit (Beyotime). The protein was separated by sodium dodecyl sulfate-polyacrylamide gel electrophoresis (SDS-PAGE) and transferred to a polyvinylidene difluoride membrane (Bio-Rad, USA). After blocking with 5% nonfat milk, the PVDF membranes were incubated with the primary antibody: Nrf2 (1:500), HO-1 (1:5,000), cleaved caspase 3 (1:1,000), Bax (1:1,000), Bcl-2 (1:1,000), β-actin (1:1,000), collagen II (1:1,000), aggrecan (1:1,000), ADAMTS-5 (1:1,000), MMP13 (1:1,000), iNOS (1:1,000), COX-2 (1:1,000), p65 (1:1,000), and IκBα (1:1,000) overnight at 4°C. Then, the membranes were washed and incubated by respective secondary antibodies at room temperature. As described in the previous study (Tang et al., 2017), the bands were detected with electro chemiluminescence plus reagent (Invitrogen), and the intensity of these bands was quantified using Image Lab 3.0 Software (Bio-Rad).

### TUNEL Method

The NP cells adhesion to the glasses was fixed by 4% paraformaldehyde for 15 min and washed by PBS for three times, and then the cells were incubated with 0.1% Triton X-100 for 10 min in 4°C as a previous study described (Chen et al., 2016). According to the manufacturer’s protocol, NP cells were stained with *in situ* Cell Death Detection Kit (F. Hoffmann-La Roche Ltd., Basel, Switzerland) for 1h. Nuclear of cells was stained with 40,6-diamidino-2-phenylindole (DAPI) after being washed for three times by PBS in the dark space to prevent fluorescence quenching. Apoptotic changes and image of apoptotic cells were observed by a fluorescence microscope (Olympus).

### Real-Time PCR

After treatment, total RNA was extracted from NP cells using TRIzol method (Invitrogen, USA). Using Prime Script-RT Reagent Kit and SYBR Premix Ex Taq (Sangon, Shanghai, China), RNA was reversed to complementary DNA (cDNA) and cDNA was amplified.The expression of target genes in different groups was measured using the ∆∆Ct method as described previously (Tang et al., 2017). The primers of COX-2 (F) 5′-GAGAGATGTATCCTCCCACAGTCA-3′ (R) 5′-GACCAGGCACCAGACCAAAG-3′; iNOS (F) 5′-CCTTACGAGGCGAAGAAGGACAG-3′, (R) 5′-CAGTTTGAGAGAGGAGGCTCCG-3′; IL-6 (F) 5′-GACAGCCACTCACCTCTTCA-3′, (R) 5′-TTCACCAGGCAAGTCTCCTC-3′; and TNF-α (F) 5′-GTCAGATCATCTTCTCGA ACC-3′, (R) 5′-CAGATAGATGGGCTCATACC-3′ were as described in a previous study (Zheng et al., 2018).

### The Measurement of NO, PGE2, TNF-α, and IL-6

After treatment, cell culture supernatants were obtained, and the NO level was measured by Griess reagent as previously described (Zheng et al., 2018). The level of PGE2, TNF-α, and IL-6 in cell culture supernatants was determined by using ELISA Kits (R&D Systems, Minneapolis, MN) according to the manufacturer’s protocol. All experiments were performed for five times.

### Safranin O–Fast Green Staining and Hematoxylin and Eosin Staining

The SD rats were sacrificed by overdosage injection of 10% pentobarbital, and the intervertebral disks of tails were harvested on 4 weeks and 8 weeks post-surgery according to the changes of rat’s MRI. The specimens were fixed in 4% paraformaldehyde more than 48 h and decalcified for more than 30 days, then dehydrated and embedded in paraffin. The sample was cut into sections (5 μm thick). Safranin O–fast green staining and hematoxylin and eosin (HE) staining, respectively, were used to assess the disk degeneration. The construction of intervertebral disk and the NP cells’ morphology and cellularity was observed by another three double-blinded experienced histology researchers using a microscope (Leica), and the histology score was evaluated according to a grading scale (Han et al., 2008; Castro-Mateos et al., 2016).

### Magnetic Resonance Imaging Method

After 4 or 8 weeks of surgery, the rats were given the MRI examination to evaluate the IDD. All rats were anesthetized by an intraperitoneal injection of 10% pentobarbital (40 mg/kg). The rats were set up in prone position for MRI, and then, the finger specific coil MRI mode was used for rats’ tail. As previous study described (Chen et al., 2016), magnetic resonance imaging was performed on all rats to evaluate the signal and structural changes in sagittal T2-weighted images using a 3.0 T clinical magnet (Philips Intera Achieva 3.0MR). The degree of IDD was evaluated by Pfirrmann grading system (Pfirrmann et al., 2001).

### X-Ray Imaging Method

After 4 and 8 weeks of surgery with or without treatment, the disk space changes of intervertebral disk were evaluated using a digital X-ray machine (Kubtec Model XPERT.8; KUB Technologies Inc.). The parameters for X-ray detection are 50 Kv and 160 μA.

### Statistical Analysis

All experiments were performed at least three times. The results were expressed as mean ± S.D. Statistical analyses were performed using SPSS statistical software program 20.0. Data were analyzed by one-way analysis of variance (ANOVA) followed by the Tukey’s test or *t* tests (and nonparametric tests) for comparison between control and treatment groups. Nonparametric data (Pfirrmann grading) were analyzed by the Kruskal–Wallis *H* test. Statistical significance was set at *P* < 0.05.

## Results

### Potential Cytotoxicity of Luteoloside and its Role as a Cytoprotectant in NP Cells

**Figure 1:**
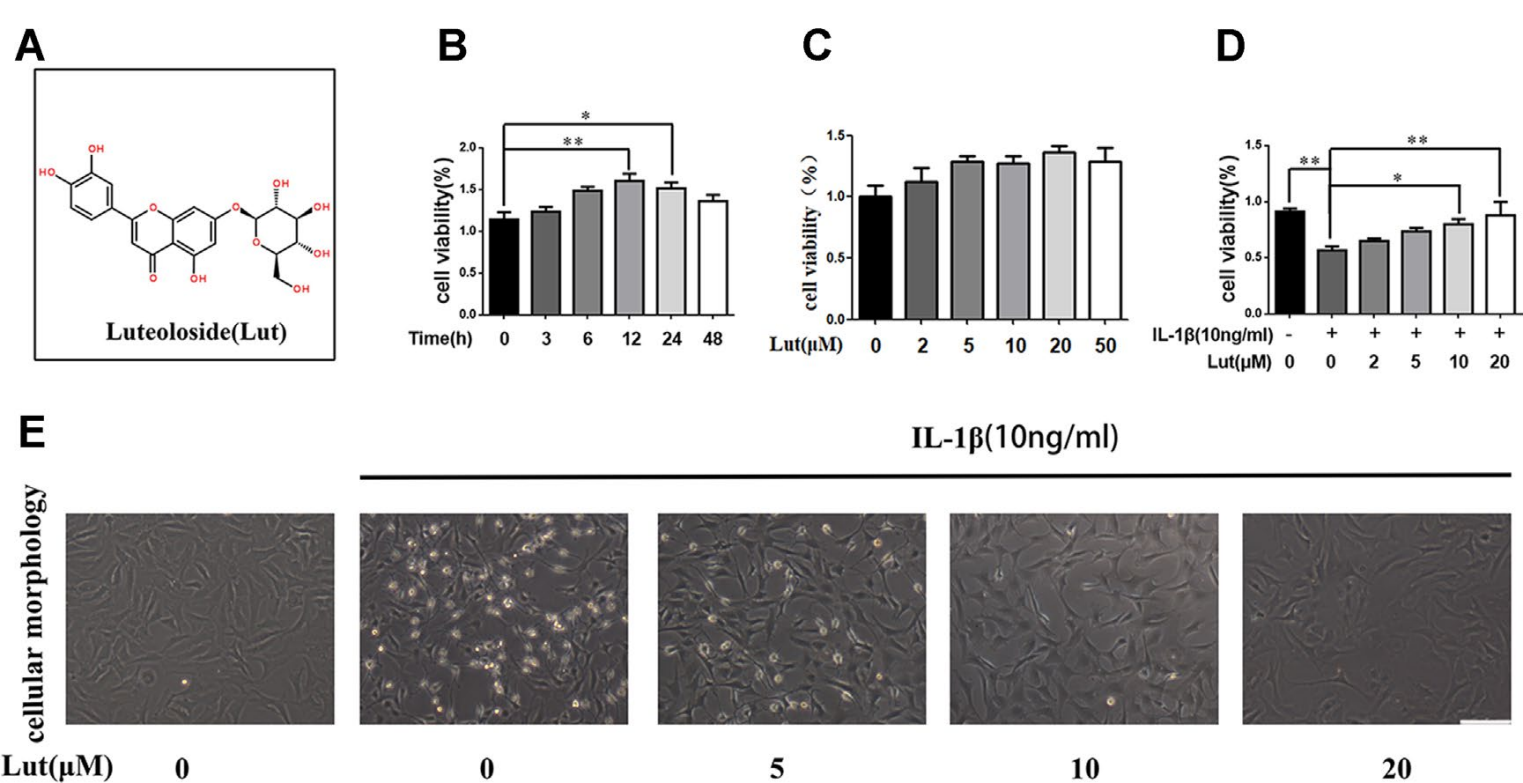
Effects of luteoloside on the cell viability of nucleus pulposus (NP) cells **(A)** Chemical structure of luteoloside. **(B)** The cytotoxic effect of 20 µM luteoloside on NP cells in a time-dependent manner using a Cell Counting Kit-8 (CCK-8) assay. **(C)** The cytotoxic effect of luteoloside on NP cells was determined at various concentrations for 24 h using a CCK-8 assay. **(D)** The cytotoxic effect of luteoloside on NP cells was determined at various concentrations with interleukin 1 beta (IL-1β) stimulation using a CCK-8 assay. **(E)** Morphological changes of NP cells treated with luteoloside in a dose dependent manner with IL-1β stimulation (original magnification × 200, scale bar: 50 µm). All experiments were performed at least three times, and the data in the figures represent the mean ± S.D. Significant differences between groups are indicated as **P < 0.01, *P < 0.05.

To determine the role of luteoloside **(** **Figure 1A** **)** in NP cells, we first investigated its time- and dose-dependent cytotoxicity in NP cells using a CCK-8 assay kit. As shown in **Figures 1B, C**, luteoloside had no significant time- or dose-dependent cytotoxic effect on NP cells. We also discovered that NP cell viability decreased during stimulation with IL-1β, but luteoloside partially reversed this phenomenon in a dose-dependent manner (**Figure 1D**; *p* < 0.01). When we exposed the NP cells to IL-1β, they shrank in size and decreased in number; this phenomenon was also partially reversed by luteoloside treatment in a dose-dependent manner (**Figure 1E**). We found that 10–20 μM was the most effective concentration of luteoloside with regard to the protection of NP cells.

### Luteoloside Treatment Protects NP Cells Against IL-1β-Induced Apoptosis

**Figure 2:**
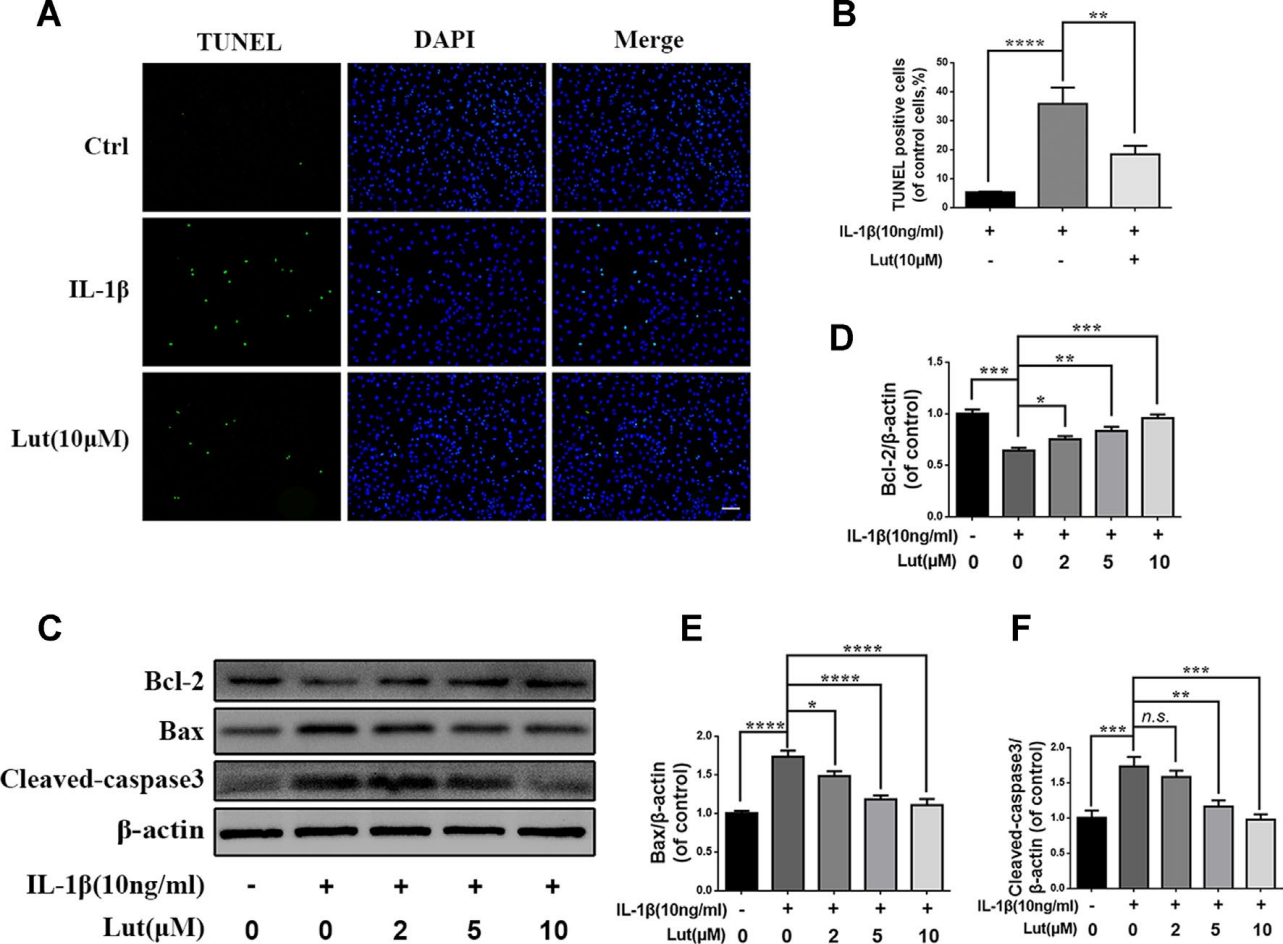
Luteoloside inhibit IL-1β induced apoptosis in nucleus pulposus cells. NP cells treated with various concentration of luteoloside for 24 h within IL-1β stimulation. **(A)** Apoptosis cells were measured in nucleus pulposus cells by terminal deoxynucleotidyl transferase dUTP nick end labeling (TUNEL) Kit (original magnification × 200, scale bar: 50 µm). **(B)** Three images were randomly selected, and the number of cells with green fluorescence was quantified. **(C)** The protein expression of cleaved-caspase 3, Bax, and Bcl-2 evaluated by western blot in nucleus pulposus cells. **(D–F)** Quantification of immunoblots of cleaved-caspase 3, Bax, and Bcl-2, and each band was normalized to each individual sample’s housekeeping gene. The experiment was repeated three times, with a representative example shown. All experiments were performed at least three times, and the data in the figures represent the mean ± S.D. Significant differences between groups are indicated as ****P < 0.0001, ***P < 0.001, **P < 0.01, *P < 0.05.

The level of IL-1β increases significantly in degenerative NP tissues, and IL-1β promotes the apoptosis of NP cells (Ito et al., 2017). In the present study, we pretreated NP cells with luteoloside and treated them with IL-1β for 24 h to simulate the inflammatory factors involved in IDD. As shown in **Figure 2A**, terminal deoxynucleotidyl transferase dUTP nick end labeling (TUNEL) indicated that NP cell apoptosis increased during IL-1β treatment, but luteoloside did partially inhibit IL-1β-induced apoptosis (**Figures 2A, B**; *p* < 0.01). A similar conclusion can be drawn from the western blot results for the NP cells. As shown in **Figure 2C**, depending on the dose, treatment with luteoloside significantly reduced the levels of cleaved caspase 3 and Bax (both pro-apoptotic proteins) and increased the level of Bcl-2 (an anti-apoptotic protein) compared to the corresponding levels in the IL-1β-treated group (**Figures 2C–F**; *p* < 0.01). In conclusion, these results show that luteoloside has a potential anti-apoptotic effect on NP cells.

### Luteoloside Regulates the Expression Levels of iNOS, COX-2, PGE2, NO, TNF-α, and IL-6 in IL-1β-Stimulated NP cells

**Figure 3:**
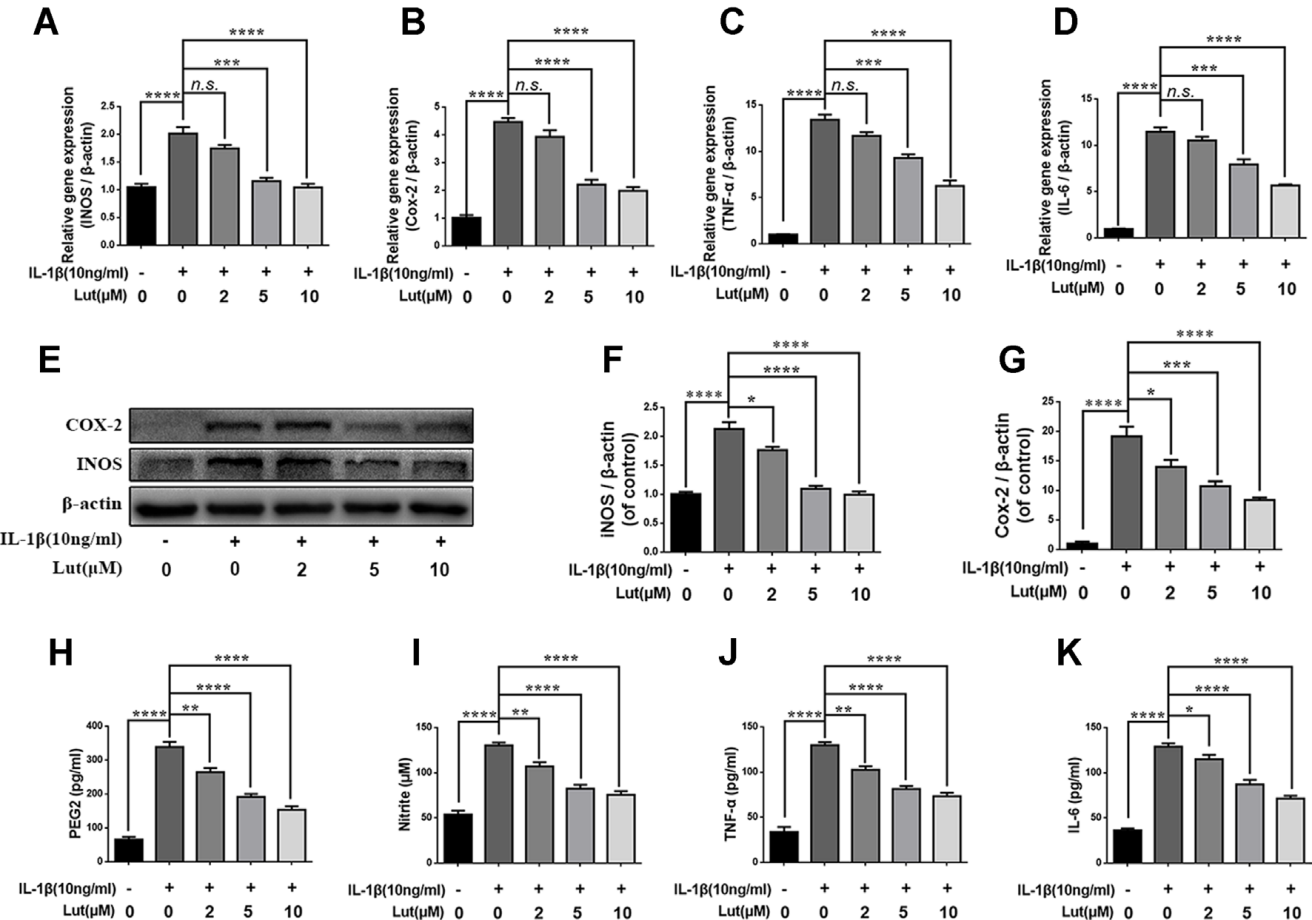
Luteoloside inhibits inflammatory response in nucleus pulposus cells. Nucleus pulposus cells treated with various concentration of luteoloside for 24 h within IL-1β stimulation. **(A–D)** The mRNA expressions of inducible nitric oxide synthase (iNOS), cyclooxygenase 2 (COX-2), tumor necrosis factor alpha (TNF-α), and interleukin 6 (IL-6) were measured by real-time Q PCR. **(E)** The protein expressions of iNOS and COX-2 in NP cells treated as above were evaluated by western blot. **(F–G)** Quantification of immunoblots of iNOS and COX-2. The experiment was repeated at least three times, with a representative example shown. **(H–K)** IL-1β-induced PGE2, nitrite, TNF-α, and IL-6 production were measured by ELISA with luteoloside in a dose-dependent manner in NP cells. All experiments were performed at least three times, and the data in the figures represent the mean ± S.D. Significant differences between groups are indicated as ****P < 0.0001, ***P < 0.001, **P < 0.01, *P < 0.05.

We investigated the effect of luteoloside on the production of inducible nitric oxide synthase (iNOS) and COX-2, which are recognized indicators of the degree of inflammation. To do so, we determined the mRNA and protein levels of iNOS and COX-2 using reverse transcription–polymerase chain reaction (RT-PCR) analysis and western blot analysis, respectively. We found that luteoloside (2, 5, and 10 μM) suppressed the expression of iNOS and COX-2 mRNA and protein during stimulation with 10 ng/ml IL-1β (**Figures 3A, B, E, F, G**). The generation of endogenous NO and PGE2 increased during IL-1β stimulation. However, depending on the dose, luteoloside suppressed the generation of PGE2 and NO (**Figures 3H, I**). RT-PCR analysis and an enzyme-linked immunosorbent assay (ELISA) also revealed that, depending on the dose, luteoloside inhibited the production of TNF-α and interleukin-6 (IL-6) (**Figures 3C, D, J, K**). These results indicate that luteoloside inhibits the generation of inflammatory mediators and cytokines in a dose-dependent manner, and the most effective concentrations are 5 and 10 μM (all *p* < 0.01).

### Luteoloside Protects NP Cells Against ECM Degradation Induced by IL-1β

**Figure 4:**
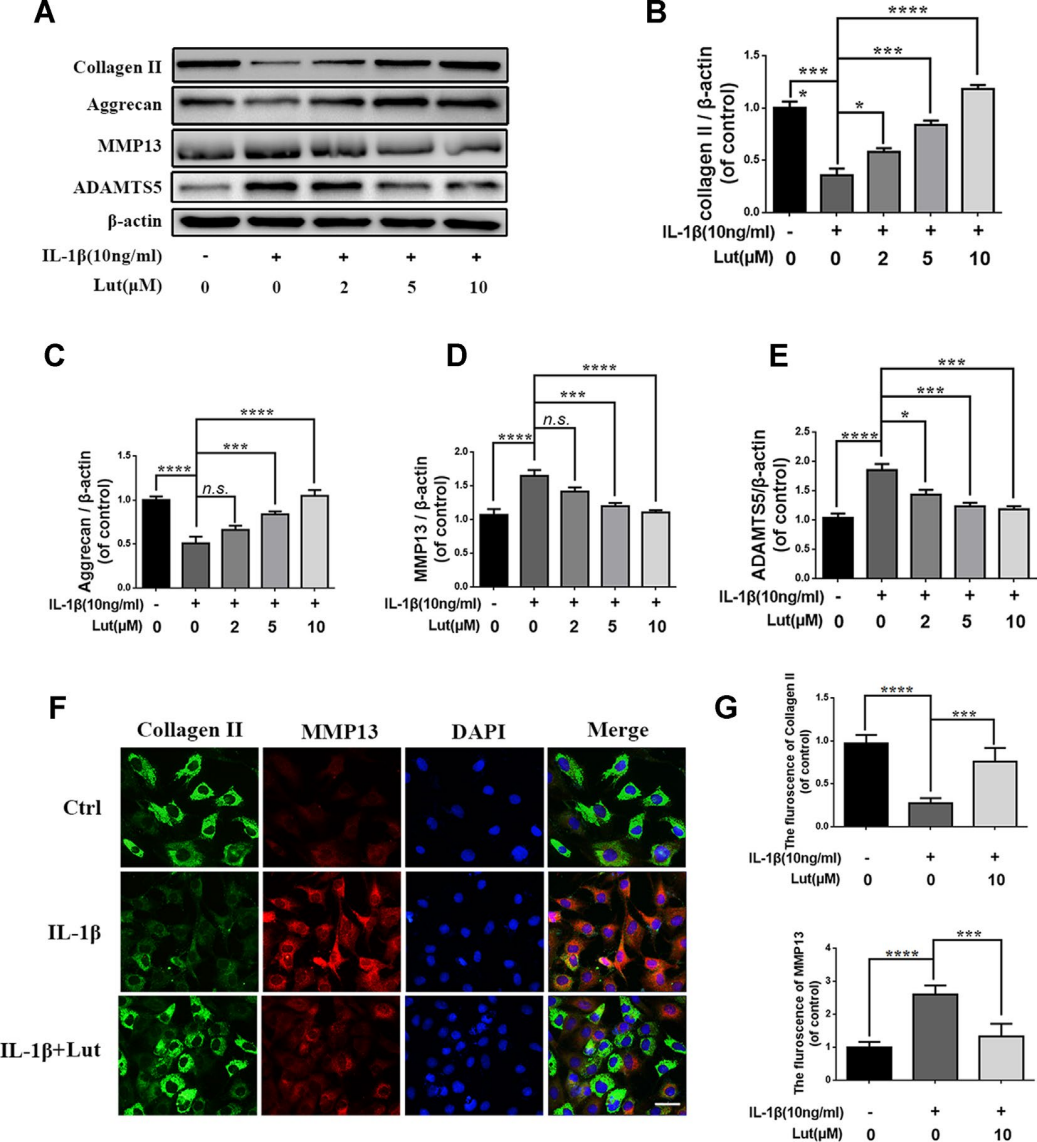
Effect of luteoloside inhibit IL-1β induced extracellular matrix degradation in nucleus pulposus cells. **(A)** Protein expressions of collagen II, aggrecan, MMP13, and ADAMTS5 in NP cells treated as above were evaluated by western blot. **(B–E)** Quantification of immunoblots of collagen II, aggrecan, MMP13, and ADAMTS5. **(F)** vThe representative collagen II (green) and MMP13 (red) were detected by the immunofluorescence combined with DAPI staining for nuclei (original magnification × 400, scale bar: 25 µm). **(G)** The fluorescence intensity of Col II (green) and MMP13 (red) was analyzed by Image J. All experiments were performed at least three times, and the data in the figures represent the mean ± S.D. Significant differences between groups are indicated as ****P < 0.0001, ***P < 0.001, *P < 0.05.

We investigated the role of luteoloside in maintaining the balance between ECM synthesis and degradation. As shown in **Figures 4A-E**, the western blot results suggest that IL-1β markedly inhibited the synthesis of collagen II and aggrecan, which are the main components of the ECM in NP cells, but promoted the production of MMP13 and ADAMTS5, which are involved in the degradation of the ECM (**Figure 4A**; *p* < 0.01). The alteration in the ECM of NP cells induced by IL-1β was reversed by luteoloside in a dose-dependent manner, especially at concentrations of 5 and 10 μM (**Figure 4A**; *p* < 0.01). Furthermore, the double immunofluorescence of collagen II (green) and MMP3 (red) revealed that these proteins mainly localized to the cytoplasm. The fluorescence intensity of collagen II decreased and that of MMP13 (a member of the MMP family) increased following IL-1β treatment. However, after treatment with luteoloside, this trend was partially reversed (**Figures 4F, G**; *p* < 0.01). Taken together, these data suggest that luteoloside protects NP cells against IL-1β-induced ECM degradation by promoting the synthesis and inhibiting the degradation of the ECM.

### Luteoloside Regulates IL-1β-Induced NF-κB Activation in NP Cells

**Figure 5:**
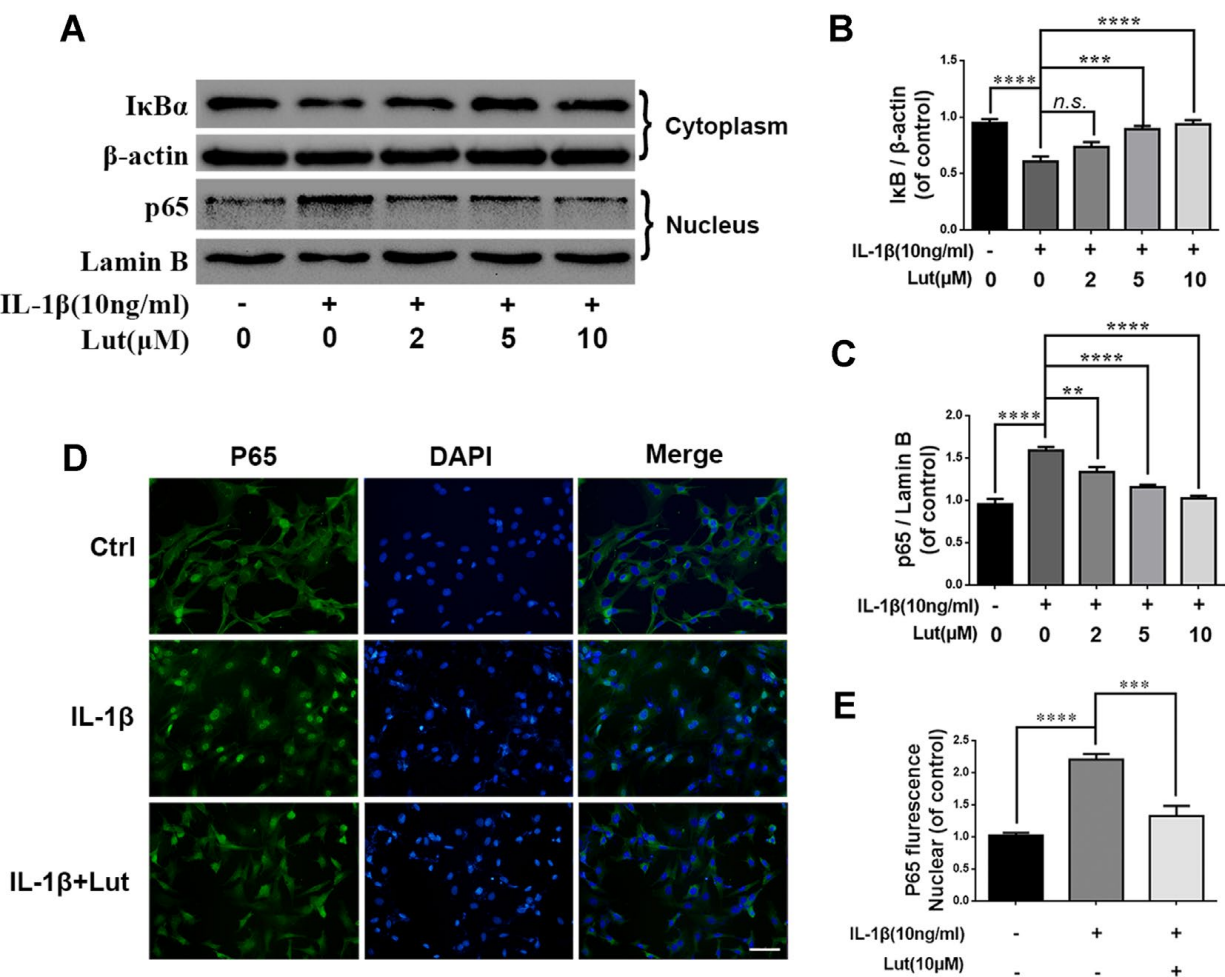
Effect of luteoloside on IL-1β-induced NF-κB activation. NP cells were pretreated with luteoloside for 24 h and then were treated with IL-1β for another 24 h. **(A)** The protein expressions of IκBα in cytoplasm and p65 in nuclear in NP cells treated as above were visualized by western blot. **(B–C)** Quantification of immunoblots of IκBα and p65. **(D)** The nuclei translocation of p65 was detected by the immunofluorescence combined with DAPI staining for nuclei (original magnification × 400, scale bar: 25 µm). **(E)** Intensity of p65 in nuclear of NP cells was quantified. All experiments were performed at least three times, and the data in the figures represent the mean ± S.D. Significant differences between groups are indicated as ****P < 0.0001, ***P < 0.001, **P < 0.01.

Several studies have shown that IDD is associated with inflammation, and NF-κB is involved in the inflammation response (Yao et al., 2016; Li et al., 2017). During IL-1β treatment, we detected several inflammation-related proteins, such as IκBα and NF-κB (p65), by western blot analysis. IκBα is an upstream target of NF-κB, and its degradation contributes to the activation of the NF-κB pathway. IL-1β treatment obviously promoted the degradation of IκBα in the cytoplasm and increased the expression of p65 in the nuclei of the NP cells (**Figures 5A-C**). As shown in **Figure 5A**, luteoloside inhibited the activation of NF-κB by inhibiting IκBα degradation in the cytoplasm and inhibiting the expression of p65 in the nuclei of the NP cells. We also used immunofluorescence staining to access the translocation of p65 during IL-1β-induced NF-κB activation in the NP cells. As shown in **Figures 5D, E**, in the absence of IL-1β stimulation, p65 is mainly located in the cytoplasm of NP cells. The fluorescence intensity of p65 increased significantly in the nuclei and decreased in the cytoplasm during IL-1β stimulation of the NP cells. This phenomenon suggests the nuclear translocation of p65 during IL-1β stimulation of NP cells. However, treatment with luteoloside markedly inhibited nuclear translocation in NP cells, as shown in **Figure 5D** and **Supplementary Figure 1**. These findings suggest that luteoloside inhibits NF-κB activation in NP cells.

### Luteoloside Regulates IL-1β-Induced NF-κB Activation and Apoptosis *via* Nrf2 Signaling in NP Cells

**Figure 6:**
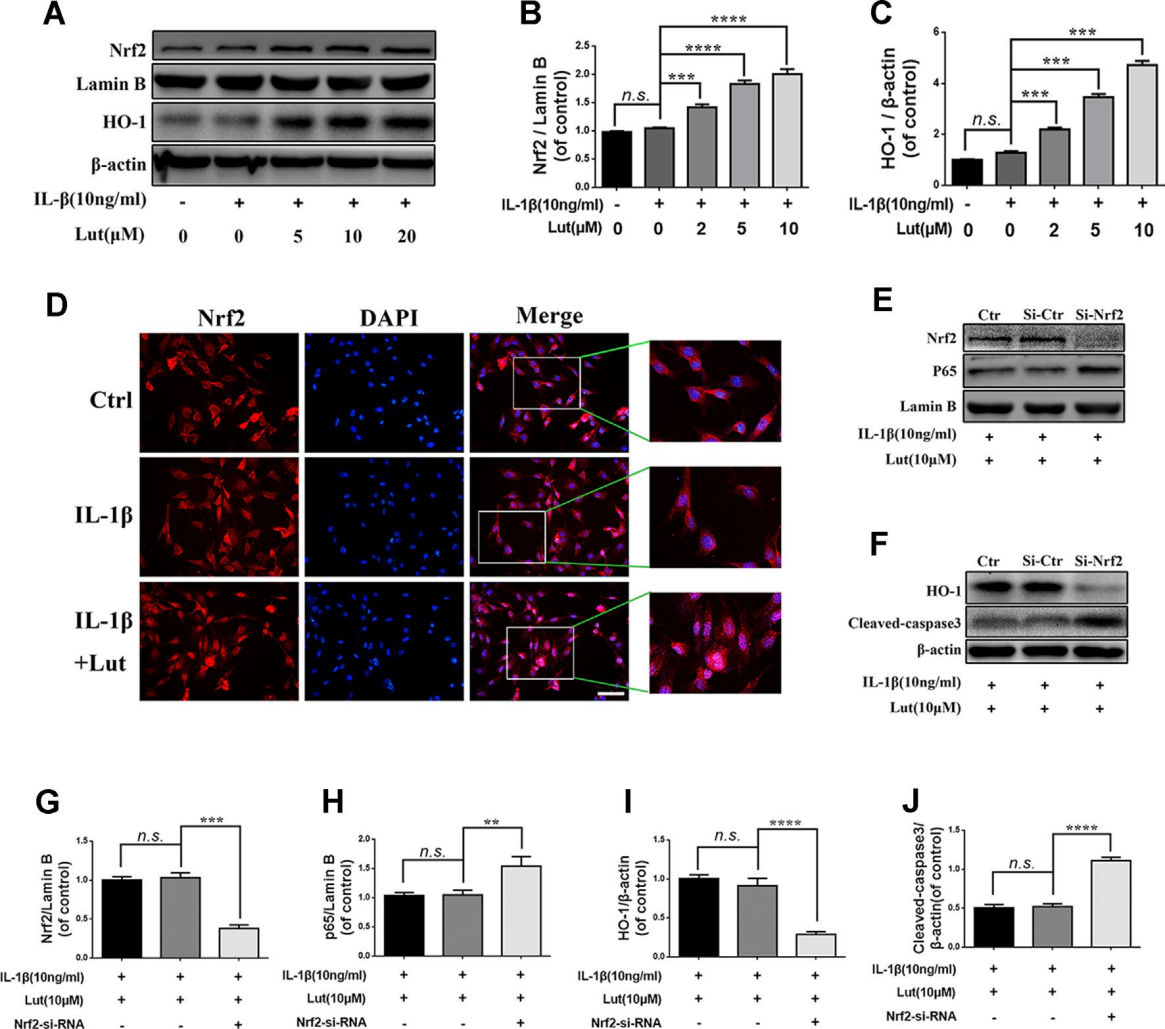
Effect of luteoloside on nuclear factor (erythroid-derived 2)-like 2 (Nrf2)/HO-1 pathway. **(A)** The protein expressions of Nrf2 in nuclear and HO-1 in cytoplasm in NP cells treated as above were visualized by western blot. **(B-C)** Quantification of immunoblots of Nrf2 and HO-1. **(D)** The nuclei translocation of Nrf2 was detected by the immunofluorescence combined with DAPI staining for nuclei (original magnification × 400, scale bar: 10 µm). The NP cells were pretreated with Nrf-2 siRNA and then cotreated with the luteoloside and IL-1β. **(E–F)** After Nrf2 knockdown, the protein expressions of Nrf2 and p65 in nuclear and HO-1 and cleaved caspase 3 in cytoplasm in NP cells treated as above were visualized by western blot. **(G–J)** Quantification of immunoblots of Nrf2, p65, HO-1, and caspase 3. All experiments were performed at least three times, and the data in the figures represent the mean ± S.D. Significant differences between groups are indicated as ****P < 0.0001, ***P < 0.001, **P < 0.01.

As shown in **Figures 6A-C** western blot analysis revealed that, depending on the dose, luteoloside increased the expression of Nrf2 in the nuclei and increased the expression of HO-1 in the cytoplasm during stimulation with IL-1β. There were no significant changes in the levels of Nrf2 and HO-1 in the pathological condition (**Figures 6A-C**; *p* > 0.05). Fluorescence staining of Nrf2 revealed that luteoloside promoted the translocation of Nrf2 into the nuclei, which was consistent with the western blot results (**Figure 6D**). As shown in **Figures 6E, G, H** western blot analysis revealed that small interfering RNA for Nrf2 (Nrf2-siRNA) inhibited the expression of Nrf2, and P65 expression was subsequently increased in the nuclei of the NP cells during stimulation with IL-1β. The expression of HO-1 was also inhibited, and the level of cleaved caspase 3 (a marker of apoptosis) increased markedly in NP cells that had been pre-transfected with Nrf2-siRNA (**Figures 6F, I, J**; *p* < 0.01). In conclusion, the Nrf2/HO-1 pathway is involved in luteoloside-induced NF-κB signaling suppression and apoptosis inhibition.

### Luteoloside Ameliorates IDD in a Puncture-Induced Rat Model

**Figure 7:**
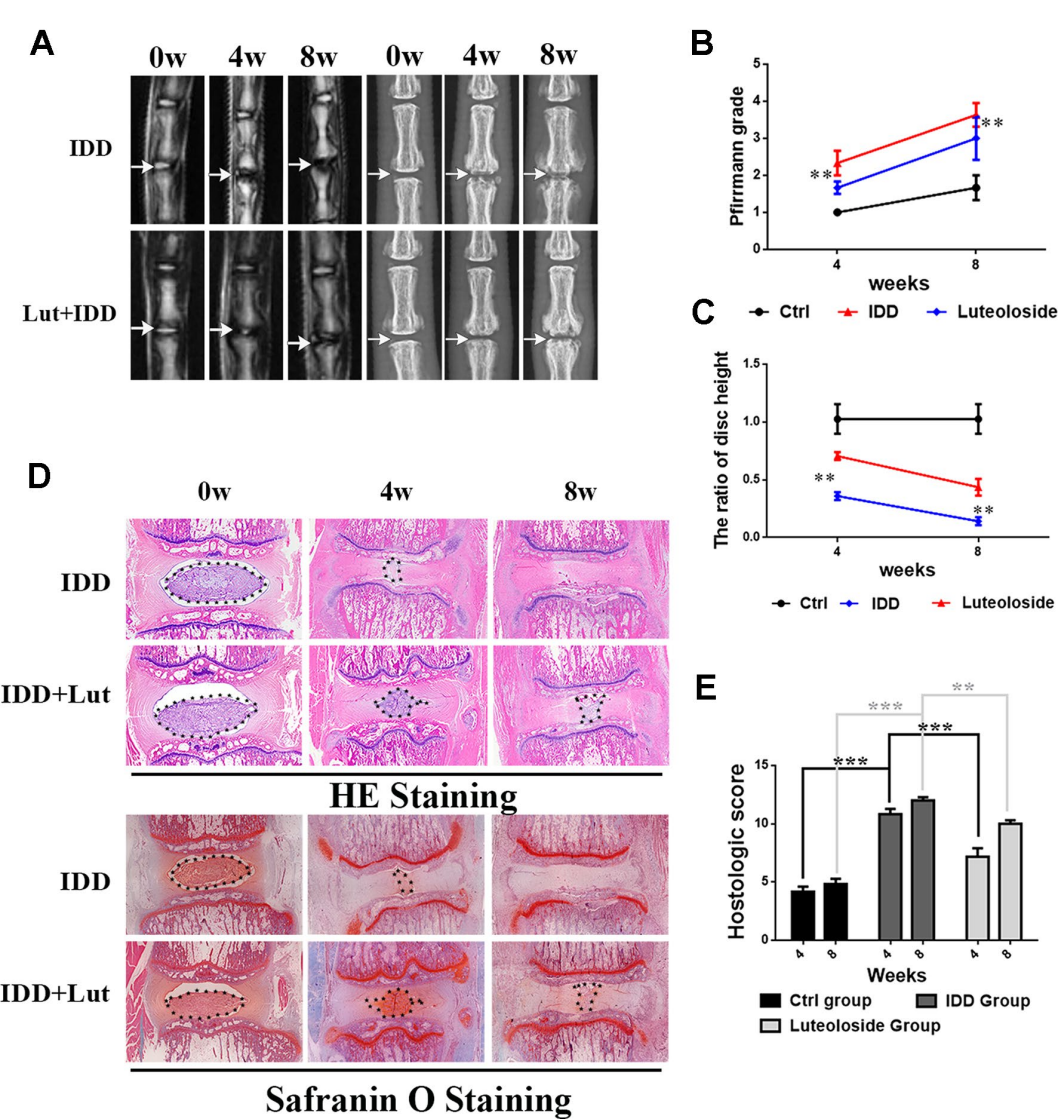
Luteoloside treatment ameliorates rat intervertebral disk degeneration (IDD) *in vivo.* Rat IDD model was established by stabbing the whole layer of annulus fibrosus (AF) through the tail skin using needles (27G) for 1 min. Luteoloside IDD group rats were injected intraperitoneally with luteoloside (10 mg/kg/day) every day. 0-, 4-, and 8-week degenerated disks were evaluated under MRI and stained with hematoxylin and eosin (HE) and safranin O (SO). **(A)** T2-weighted MRI of a rat tail with a needle-punctured disk at 4 and 8 weeks post-surgery (white arrow: location of the needle-puncture disk). Digital X-ray image of intervertebral disk from different experimental groups (white arrow: location of the needle-puncture disk). **(B)** The Pfirrmann MRI grade scores in three groups at week 4 and week 8 (six rats at each time point for each group). **(C)** Quantification of narrowing of disk space. **(D)** Representative HE staining and SO staining of disk samples from different experimental groups at 4 weeks and 8 weeks post-surgery (original magnification × 40, scale bar: 100 µm). Three sections were randomly selected for quantification, with a representative example shown. **(E)** The histological grades evaluated at 4 weeks and 8 weeks post-surgery in three groups (six rats per group). All experiments were performed at least three times, and the data in the figures represent the mean ± S.D. Significant differences between groups are indicated as ***P < 0.001, **P < 0.01.

We established a rat model of IDD by puncture induction to evaluate the effects of luteoloside on IDD *in vivo*. The degree of IDD in the rats was assessed by magnetic resonance imaging (MRI) and quantified by Pfirrmann MRI grade scores. As shown in **Figures 7A, B**, the MRI images obtained 4 weeks after puncture revealed that the punctured disks were inhomogeneous, with an intermittent gray signal intensity, but the results do show hyperintense white signals in the luteoloside-treated group compared to the untreated IDD group. The results were similar after 8 weeks (**Figures 7A, B**). Furthermore, the Pfirrmann MRI grade scores, which indicate the degree of disk degeneration, were significantly lower in the luteoloside group than in the saline group at 4 weeks (*p* < 0.01) and 8 weeks (*p* < 0.01) (**Figure 7B**). Moreover, the X ray results also confirm the hypothesis described above. We found that the height of the intervertebral space was significantly lower in the IDD group than in the luteoloside treatment group at both 4 and 8 weeks after surgery (*p* < 0.01; **Figures 7A, C**).

As shown in **Figures 7D, E**, HE staining (used to examine the general histological structure of NP tissues) revealed that the structure of the NP had almost disappeared in the vehicle (saline)-treated IDD group at the 4-week time-point. However, the structure was preserved in the luteoloside-treated IDD group, and the well-organized shape of the NP tissues was still clearly visible within the disks, which almost disappeared in the vehicle group. Safranin O (SO), which stains proteoglycans and glycosaminoglycans, revealed that both the structure and the ECM of the NP tissues were better preserved in the luteoloside-treated group. At the 8-week time-point, HE and SO staining revealed that the effects of luteoloside on the morphological and ECM preservation of NP were much weaker than at the 4-week time-point; only a small part of the NP was preserved in the luteoloside treatment group, but there were still differences between the luteoloside and vehicle groups, suggesting that luteoloside also ameliorate IDD during long-term (8-week) treatment.

**Figure 8:**
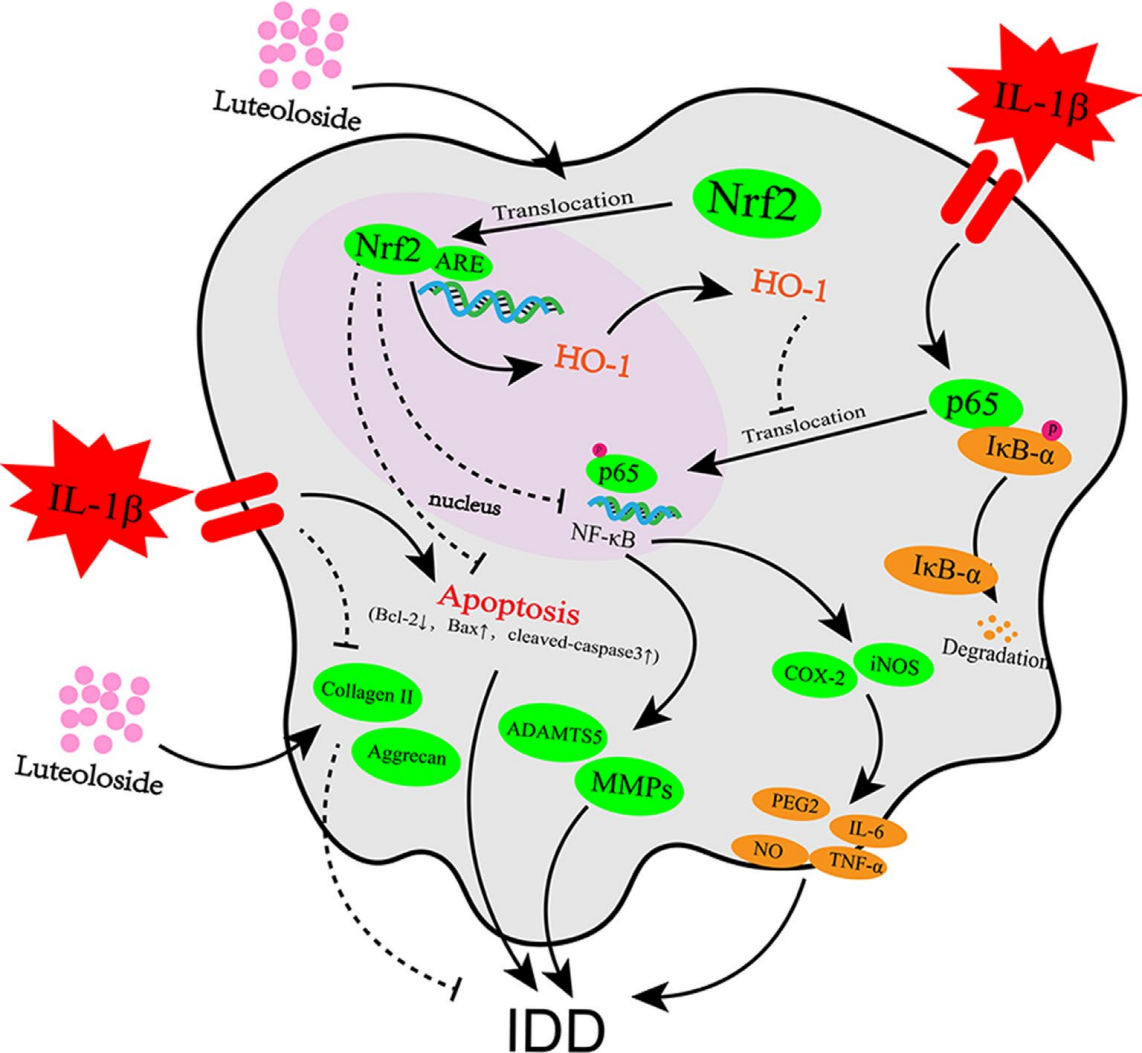
Potential molecular mechanism involved in luteoloside treatment on NP cells. IL-1β stimulation promotes inflammation response, apoptosis, and extracellular matrix (ECM) degradation through activation of NF-κB signaling pathway. Luteoloside treatment attenuated IL-1β-induced apoptosis through Nrf2 activation and attenuated inflammation response and ECM degradation in NP cells *via* suppressing NF-κB activity by Nrf2/HO-1 pathway.

## Discussion

Surgery, which is one of the major treatments for IDD, can only relieve pain temporarily and cannot eradicate the causes of IDD. Moreover, there is no effective way to treat degeneration apart from with surgery. In the present study, we investigated the protective effects of luteoloside on the IL-1β-induced inflammation response, ECM degradation, and apoptosis in NP cells *in vitro*. We reported the protective effects of luteoloside in NP cells *via* the Nrf2/HO-1 signaling axis (**Figure 8**) and an *in vivo* study using a puncture-induced rat model indicated that luteoloside ameliorates the progression of IDD.

Inflammation and apoptosis are considered major mediators of IDD pathogenesis (Yang et al., 2018; Zheng et al., 2018), and IL-1β is one of the most important inflammation cytokines that contribute to IDD (Risbud and Shapiro, 2014). Therefore, we chose IL-1β to stimulate the NP cells and found that it activated the NF-κB signaling pathway, triggered the inflammation response, and promoted the apoptosis of NP cells and the degradation of the ECM. The increase of NP cell apoptosis caused the leakage of NP tissues and reduced the synthesis of collagen II and aggrecan, thereby accelerating IDD (Chen et al., 2016). The suppression of excessive NP cell apoptosis could ameliorate the progression of disk degeneration (Zhao et al., 2007). Caspase 3 is an apoptotic protein. Its knockdown could suppress the generation of matrix-degrading enzymes (e.g., MMP-3, MMP-13, ADAMTS-4, and ADAMTS-5) and promote the expression of pro-anabolic proteins (e.g., tissue inhibitor of metalloproteinases 1, collagen II, and aggrecan) (Cheng et al., 2018). Taken together, these studies, including the present investigation, suggest that the inhibition of NP cell apoptosis balances the synthesis and degradation of the ECM and ameliorates IDD.

Furthermore, previous studies have revealed that activation of the NF-κB signaling pathway is involved in the pathophysiological progression of IDD (Kang et al., 2017; Li et al., 2017). The NF-κB signaling pathway mediates the production of proteinases and pro-anabolic proteins—such as aggrecan and collagen II—in NP cells (Wang et al., 2018). IL-1β stimulation triggers the phosphorylation of IκBα, which consequently frees and translocates p65 from the cytoplasm to the nucleus. IκBα is subsequently degraded in the cytoplasm. NF-κB is highly activated in the nucleus in diverse diseases. It promotes the transcription of proinflammatory cytokines, chemokines, adhesion molecules, MMPs, Cox-2, and iNOS, which subsequently contribute to the degradation of the ECM (Tak and Firestein, 2001; Tang et al., 2017). The present study revealed that luteoloside inhibits NF-κB and consequently reduces the production of inflammatory mediators, cytokines, and matrix-degrading proteases—such as members of the MMP family—*via* the NF-κB signaling pathway (*p* < 0.01) (**Figures 4A** and **5A**).

Numerous recent studies have demonstrated that Nrf2 plays a vital role in the regulation of inflammation responses in osteoarthritis (OA), and its absence is accompanied by an increase in susceptibility to inflammatory disorders (Kensler et al., 2007; Tang et al., 2017; Khan et al., 2018). Nrf2-deficient cells have increased p65-NF-κB protein levels, and Nrf2 has an antagonistic effect on the NF-κB pathway (Cuadrado et al., 2014). Studies have shown that the activation of Nrf2 suppresses the NF-κB signaling pathway and ameliorates the progression of OA (Tang et al., 2017; Zheng et al., 2018). A recent study showed that Nrf2/HO-1 activation protects the human umbilical vein endothelium from microcystin-LR-mediated apoptosis (Shi et al., 2018). Nrf2 overexpression in OA chondrocytes promotes the expression of anti-apoptotic proteins and inhibits the expression of pro-apoptotic proteins (Khan et al., 2018). NP and cartilage share a common developmental lineage at the molecular level, which is the main functional component of the intervertebral disk and consists of small chondrocyte-like cells with typical chondrocyte-like morphology (Wang et al., 2018). Thus, Nrf2 may also affect the progression of IDD. Therefore, we investigated the role of Nrf2 in NP cells by using Nrf2-siRNA to suppress the expression of Nrf2. The results corroborated our hypothesis (**Figures 6E, F**). Taken together, our data demonstrate that Nrf2 ameliorates inflammation by suppressing the NF-κB signaling pathway and anti-apoptotic function and acts *via* activation of the Nrf2/HO-1 signaling axis in NP cells.

NF-κB activation is involved in the initiation of apoptosis in inflammatory diseases (Tak and Firestein, 2001). Although the role of NF-κB is not always straightforward, luteoloside may protect NP cells against apoptosis and the inflammation response by suppressing NF-κB, because many studies have suggested that there is potential cross-talk between the Nrf2 and NF-κB pathways (Li et al., 2008; Bellezza et al., 2010; Cuadrado et al., 2014; Wardyn et al., 2015). Studies have shown that persistent cardiac myocyte NF-κB p65 activation associated with heart failure exacerbates cardiac remodeling by aggravating pro-inflammatory, pro-fibrotic, and pro-apoptotic effects (Hamid et al., 2011). Furthermore, NF-κB transcriptionally regulates the expression of several members of the Bcl-2 gene family (Bcl-2 is a typical anti-apoptotic protein), and cell apoptosis is associated with the phosphorylation of NF-κB p65 (Ji et al., 2017). In the present study, luteoloside also inhibited the translocation of nuclear p65, suppressed apoptosis-related proteins, and promoted the expression of anti-apoptotic proteins (**Figures 2C** and **5A, D**). Therefore, luteoloside may protect NP cells against apoptosis and the inflammation response by inhibiting NF-κB signaling or activating the Nrf2/HO-1 axis.

The MRI data obtained in the present study using a puncture-induced rat model of IDD suggested that intraperitoneal injections of luteoloside inhibit IDD and attenuate the downregulation of collagen II and aggrecan. These *in vivo* results further confirm the protective effects of luteoloside with regard to IDD. To make the efficacy of luteoloside more convincing *in vivo*, it needs to be verified in other IDD models (Choi et al., 2018; Wang et al., 2018; Ao et al., 2019), which will be the direction of our next research.

In conclusion, the present study demonstrates that luteoloside protects NP cells by suppressing key molecular events involved in inflammation, apoptosis, and matrix degradation in NP cells and tissues. Our results also demonstrate that the protective effects of luteoloside in NP cells arise through activation of the Nrf2/HO-1 signaling axis. Luteoloside is a natural therapeutic agent that can be used to treat inflammation-related IDD. Our study provides novel information about the action of Nrf2, which is a promising candidate for the development of new therapies for the management of IDD.

## Data Availability

The raw data supporting the conclusions of this manuscript will be made available by the authors, without undue reservation, to any qualified researcher.

## Ethics Statement

All surgical interventions, treatments and postoperative animal care procedures were performed in strict accordance with the Animal Care and Use Committee of Wenzhou Medical University (wydw2014-0129).

## Author Contributions

XYW, XZ, and YL contributed to the conception of the study; JL contributed to the revision of the manuscript and provided important intellectual and technical contribution; JC and ZZ contributed significantly to analysis and manuscript preparation; TX, ZS, XBW, and YD performed the cell experiment and data analyses and wrote the manuscript; NT and HJ performed the animal experiment and data analyses and wrote the manuscript; and SS and WG helped perform the analysis with constructive discussions.

## Funding

This work was supported by a grant from the Zhejiang public service technology research program/social development (LGF18H060008), Zhejiang Provincial Natural Science Foundation of China (LY17H060010 and LY18H060012), major scientific and technological project of medical and health in Zhejiang Province (WKJ-ZJ-1527), Zhejiang Province medical science and technology project (2017171281), Wenzhou Bureau of Science and Technology project (Y20160136), and Zhejiang Undergraduate Talent Project (2016R413072).

## Conflict of Interest Statement

The authors declare that the research was conducted in the absence of any commercial or financial relationships that could be construed as a potential conflict of interest.
