# Supplementary material for: Luteoloside Inhibits IL-1β-Induced Apoptosis and Catabolism in Nucleus Pulposus Cells and Ameliorates Intervertebral Disk Degeneration
Source: Front Pharmacol. 2019 Aug 5;10:868. doi: 10.3389/fphar.2019.00868 (PMC6690034; doi:10.3389/fphar.2019.00868)
Supplement: Supplementary file 1 [file DataSheet_1.docx]

**Supplementary materials**

**Supplementary Figure 1**

**
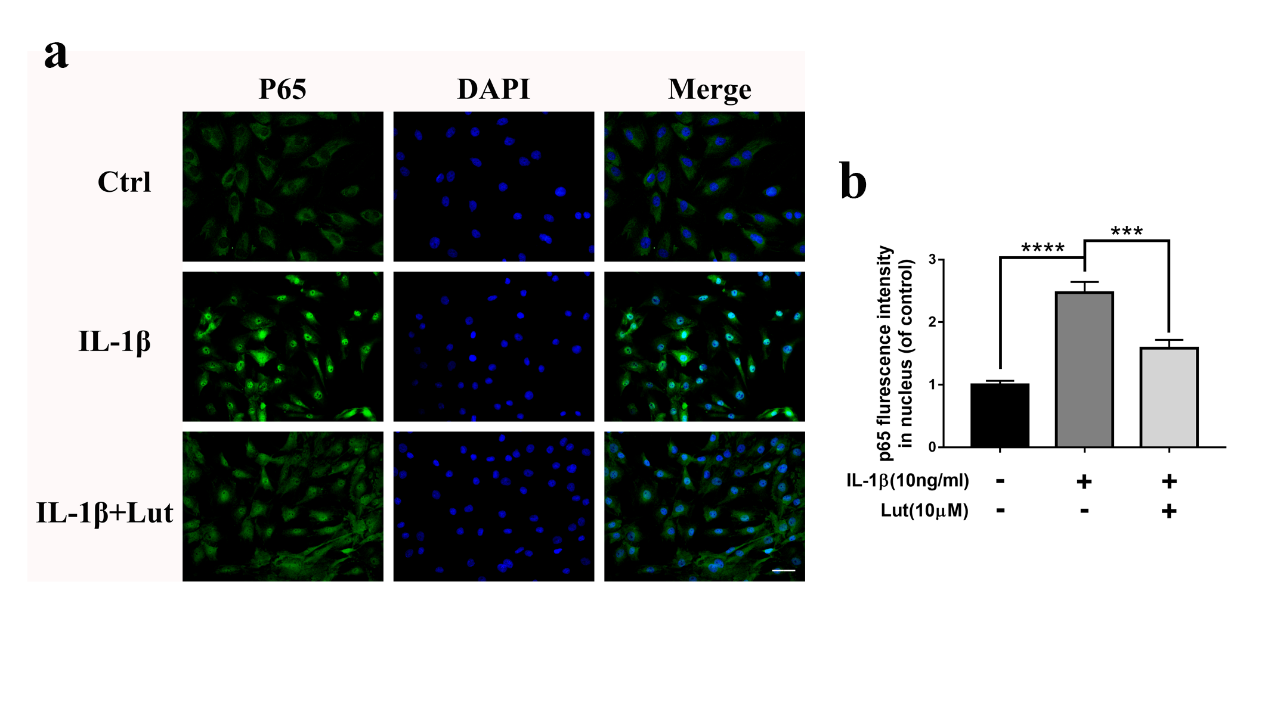
**

**Supplementary Figure 1:** **Luteloside treated NP cells after IL-1β treatment.** (a) The nuclei translocation of p65 was detected by the immunofluorescence combined with DAPI staining for nuclei (original magnification × 400, scale bar: 20 μm). (b) Intensity of p65 in nucleus of NP cells was quantified. The experiment were performed at least three times and the data in the figure represent the mean ±S.D. Significant differences between groups are indicated as**** P＜0.0001, *** P＜0.001, **P＜0.01, *P＜0.05
